# Hospitals and the Spirit Tax

**Published:** 1917-03-10

**Authors:** F. A. Hocking

**Affiliations:** The Dispensary, London Hospital


*To the Editor of* THE HOSPITAL.

### Hospitals and the Spirit Tax.

*To the Editor of* THE HOSPITAL.

SIR,—May I appeal to your sense of fairness to publish this letter?

(1) *As users of alcohol* hospitals are in receipt of two payments from the Government. They are (*a*) an "allowance" and (*b*) a "rebate."

These terms refer to quite different transactions, and are the terms officially used by the Board of Customs and Excise, which arranges the payments.

(2) Hospitals *as hospitals* will receive a third payment, which is called by the Local Government Board a "grant in relief."

These words were used in the terms of reference issued to the Treasury Committee appointed to consider the question, and it was the term used, in an extended form, by the Committee throughout its deliberations, as well as in a circular-letter sent to a large number of hospitals in London and the provinces.

Whatever anyone may think of the suitability or otherwise of the above three terms, they can only be used in the manner designated by the Government Departments. To use them in any other sense leads to hopeless confusion. My knowledge of the method of allocation recommended by the Committee renders the term "grant in relief" particularly suitable.

Someone has committed an indiscretion in communicating to the Press, directly or indirectly, some remarks made in the course of a private conversation, and has aggravated the indiscretion by inaccuracy which fully warrants my use of the word "travesty." The relevance of the reference to the President of the Incorporated Association of Hospital Officers is not apparent to me.—Yours faithfully, F. A. HOCKING.

The Dispensary, London Hospital,
March 3, 1917.

[We publish this letter, but it does not alter the facts.—ED. THE HOSPITAL.]

